# Lava Lamp-Induced Methemoglobinemia in Dementia: A Case Report

**DOI:** 10.7759/cureus.29441

**Published:** 2022-09-22

**Authors:** Thomas G Powell, Eric Choi, Srinivas Vuppala, Hafez Hayek

**Affiliations:** 1 Osteopathy, Campbell University, Conway, USA; 2 Family Medicine, Conway Medical Center, Conway, USA; 3 Internal Medicine, Conway Medical Center, Conway, USA; 4 Critical Care, Conway Medical Center, Conway, USA

**Keywords:** airway emergency, dementia, dementia safety, methylene blue, ascorbic acid, methemoglobin, methemoglobinemia

## Abstract

An elderly patient with progressive dementia presented with nonspecific symptoms of fatigue, skin discoloration, shortness of breath, and altered mental status. She quickly developed respiratory failure requiring emergent endotracheal intubation. Initial laboratory results revealed methemoglobinemia levels greater than 30% with blood appearing black in hue. The etiology of her acute symptoms and the inciting substance of the disease was an ongoing discussion with the patient’s family, which ultimately revealed accidental ingestion of lava lamp fluid as the cause. Although rare, methemoglobinemia is a medical emergency requiring prompt diagnosis and treatment. When a thorough history fails to reveal a possible source, alternative origins should be investigated, such as household products.

## Introduction

Methemoglobinemia is a congenital or acquired condition where circulating hemoglobin denudes its oxygen-carrying capacity, resulting in hypoxia without hypoxemia. In the disease, the reduced form of iron, ferrous, assumes an oxidized state, ferric, leaving hemoglobin’s oxygen capacity severely diminished [1]. While the congenital form is rare, there are numerous acquired forms of the disease with most reports being pharmacologic [1-3]. However, ingestion of toxic substances remains a commonly reported cause of methemoglobinemia, such as paint thinners or antifreeze [4,5]. These compounds often contain nitrites and nitrates, known antecedents of the disease [4,5]. A literature review demonstrated a single previously reported case of methemoglobinemia induced by ingesting lava lamp contents [4]. We present a similar case of methemoglobinemia induced by accidental ingestion of lava lamp liquid successfully treated with second-line therapy.

## Case presentation

**Figure 1:**
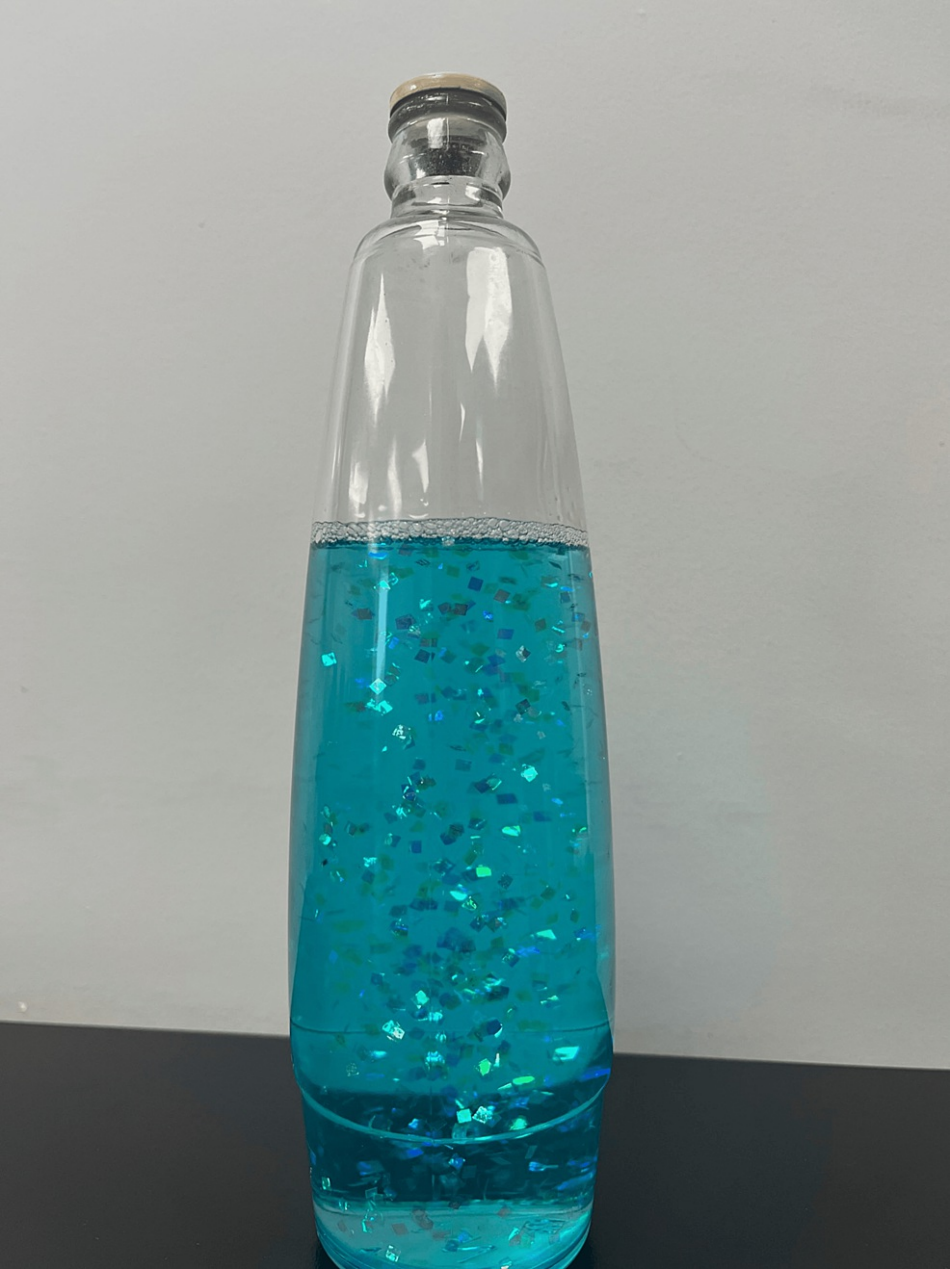
Partially consumed lava lamp recovered from the patient’s home

An 88-year-old female with a history of advanced dementia and breast cancer status-post bilateral partial mastectomy presented with blue-pale skin coloration, mild hypoxemia, and encephalopathy. Her husband provided all the history due to her altered mental status. He stated the night prior to presentation, she handed him a bottle, which he opened and returned to her without verifying its contents (Figure 1). Overnight, she began coughing with three episodes of post-tussive emesis. The following morning, she was found to be a pale-blue color and hypoxic with an altered mental status by emergency medical services. 

Upon presentation to the emergency room, she continued to be hypoxic on room air with oxygen saturations at 86%-88%, which increased to 92% with 3 L of oxygen by nasal cannula. The remainder of her physical examination included a blood pressure of 104/65, a heart rate of 94 beats per minute, and a respiratory rate of 21 breaths per minute. A Glasgow Coma Scale was found to be 6 at this time.

Routine laboratory results at presentation were normal, except for the following: white blood cell count of 16.4 × 10^9^/L, glucose of 164 mg/dL, creatinine level of 1.27 mg/dL, chloride level of 114 mEq/L, carbon dioxide level of 24 mmol/L, anion gap of 2 mmol/L, calcium level of 13.6 mg/dL, lactic acid of 3.9, and magnesium level of 1.5 mg/dL. Urinalysis revealed no signs of pyuria, proteinuria, or hematuria. A 12-lead electrocardiogram showed normal sinus rhythm, normal axis, and no sign of ischemia. A chest radiograph was read as subtle bibasilar alveolar densities likely secondary to atelectasis. A computed tomography (CT) scan of her chest demonstrated ground-glass airspace disease in the lingula. A CT scan of her head demonstrated no acute intracranial abnormalities. Treatment for presumed aspiration pneumonia was initiated based on clinical and radiological findings with ampicillin-sulbactam, which was discontinued upon further diagnosis confirmation.

**Figure 2:**
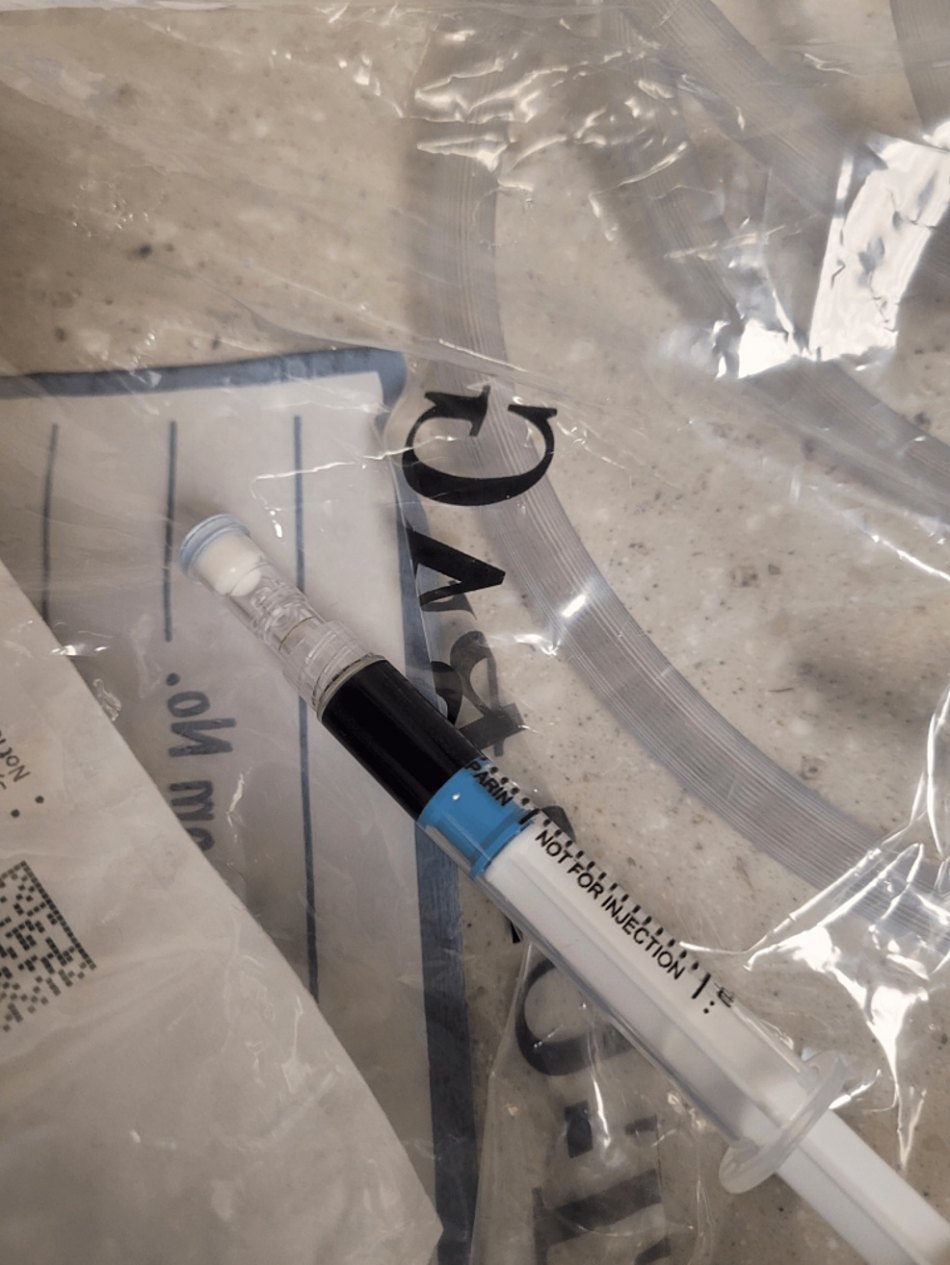
Initial arterial blood gas demonstrating blood that is black in hue

She was transferred to the intensive care unit and subsequently intubated for deteriorating respiratory status. Arterial blood gas (ABG) following intubation demonstrated a pH of 7.26, partial pressure of carbon dioxide (PaCO_2_) of 38.7 mmHg, partial pressure of oxygen (PaO_2_) of 211 mmHg, and a methemoglobin level over 30%, which is the maximum read for our facility. Visualization of the ABG sample was black in coloration (Figure 2).

**Figure 3:**
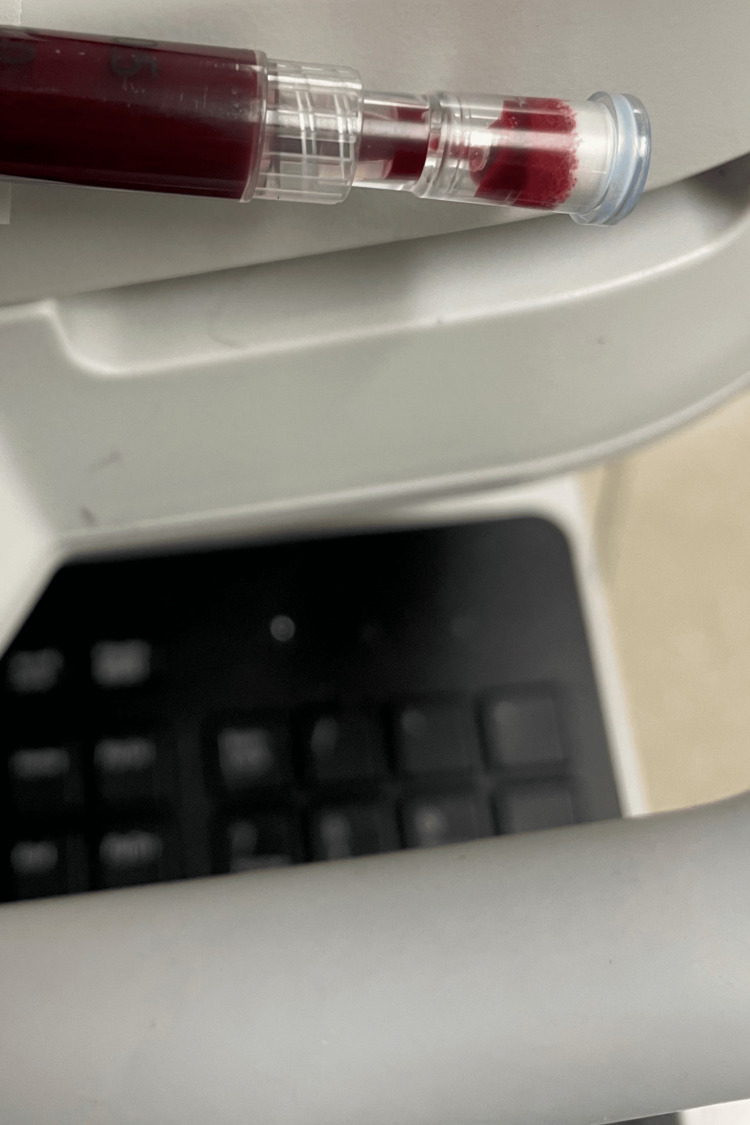
Final arterial blood gas following ascorbic acid treatment demonstrating blood with normal color

We promptly began treatment with ascorbic acid 5 g and obtained an ABG every six hours. She received a total of four doses within 24 hours. Six hours after the first dose, her methemoglobin level was 23.8%; after the second dose, it was 10.3%; and after the third dose, it was 2.9%. Final ABG demonstrated blood with normal color (Figure 3). A fourth dose was provided after consulting poison control. Calcium levels normalized throughout treatment as well. The next morning, her skin discoloration improved while also maintaining her oxygen saturations consistently above 98%. She was promptly extubated and continued to maintain an adequate respiratory status. She remained delirious and became a harm to herself for several days, requiring multiple doses of haloperidol following treatment completion. After three days, her delirium improved; however, her family reported that she did not return to her baseline cognitive functional level.

## Discussion

This is a case of an 88-year-old female with advanced dementia diagnosed with methemoglobinemia and survived after the presumed ingestion of lava lamp contents. Unfortunately, chemical analysis of the fluid was unable to be performed to aid in toxin identification in this case. Our patient presented with a clinical picture consistent with aspiration pneumonia, which was previously described in a similar case by Funke et al. [4]. Our diagnosis of methemoglobinemia was confirmed with an ABG. The clinical presentation of methemoglobinemia demonstrates hypoxia without hypoxemia that is typically unresponsive to supplemental oxygen. The diagnosis is classically supported by the presence of chocolate-colored blood. Interestingly, our patient was saturating well on low oxygen requirements at presentation but did experience airway compromise, likely secondary to severe metabolic encephalopathy superimposed on underlying dementia. The case presented by Funke et al. also demonstrated rapid respiratory deterioration [4]. Physicians must have a high clinical concern for respiratory compromise in these patients, especially elderly patients with comorbidities. We advise obtaining an ABG when there is a high clinical concern for methemoglobinemia or respiratory compromise of unknown cause.

Methylene blue is the typical antidote when methemoglobin concentrations are above 30% or in the presence of significant symptomatology [2]. The medication is within the monoamine oxidase inhibitor class of medications and therefore should be avoided in the presence of other serotonergic agents due to the risk of inducing serotonin syndrome [6]. Our patient was taking home duloxetine and trazodone, which are major contraindications for the administration of methylene blue [6]. The recommended treatment when methylene blue is contraindicated consists of ascorbic acid at high doses, hyperbaric oxygen therapy, or transfusion therapy [1]. The efficiency of ascorbic acid in treatment is well established, but there are no guidelines on dosing or monitoring. Mohammed et al. consolidated several case reports with ascorbic acid as the primary treatment at varying doses and frequencies [7]. Our treatment regimen was similar to their dosing frequency but with a larger dose. The drop observed with our first dose can not be quantified due to restrictions of measuring upper limits of methemoglobin levels due to facility restrictions. In our case, a decline of 13.5% occurred after our second dose. This is comparable to the decrease of 12.2% they observed [7]. More research is required to define the required dose and frequency of ascorbic acid when indicated and the expected correction of methemoglobin level.

In our case, we had a delay in determining the etiology of methemoglobinemia due to multiple factors including severe metabolic encephalopathy superimposed on dementia and the patient’s husband being a relatively poor historian. On presentation, an extensive review of the patient’s home medication list revealed no direct etiology for methemoglobinemia. A follow-up examination of her husband’s medications revealed the same. The remainder of her family then performed a thorough investigation of their home, in which they discovered a partially consumed lava lamp container next to their bed. It is crucial to consider unlikely causes of the disease in patients with baseline dementia or if the etiology is not immediately apparent. We encourage investigation of all medical, environmental, and supplemental etiologies in these cases.

A retrospective cohort study demonstrated how there is a double risk of unintentional poisoning for patients with Alzheimer’s disease [8]. This presents a unique challenge for clinicians when managing home safety. For this reason, it is vital in the setting of acquired methemoglobinemia that families be counseled on specific strategies to decrease overall at-home injury risk. Strategies to decrease this risk include the involvement of professional caregivers, safe medication storage options, or the removal of harmful substances and items from the home completely [8]. It is crucial for clinicians to coordinate proper disposition planning in these patients to ensure safety.

## Conclusions

The literature review suggests that our case is the second reported incident of methemoglobinemia induced by the consumption of lava lamp contents. This disease classically demonstrates continued hypoxia regardless of supplemental oxygen, which our case did not display. Rapidly progressive respiratory decline should be accounted for with this disease in elderly patients, especially those with comorbidities. Our patient was treated with ascorbic acid instead of methylene blue due to the risk of serotonin syndrome in the presence of serotonergic medications, duloxetine, and trazodone. She responded well to treatment with cessation of respiratory support within one day. Our case demonstrates the importance of persistence when investigating likely etiologies including considering unlikely sources. Finally, accidental poisoning presents a health risk challenge to clinicians that necessitates the implementation of proper safety precautions.
